# Beyond traditional orthopaedic data analysis: AI, multimodal models and continuous monitoring

**DOI:** 10.1002/ksa.12657

**Published:** 2025-03-22

**Authors:** Felix C. Oettl, Bálint Zsidai, Jacob F. Oeding, Michael T. Hirschmann, Robert Feldt, Thomas Tischer, Kristian Samuelsson, ESSKA Artificial Intelligence Working Group

**Affiliations:** ^1^ Department of Orthopedic Surgery, Balgrist University Hospital University of Zürich Zurich Switzerland; ^2^ Hospital for Special Surgery New York New York USA; ^3^ Department of Orthopaedics, Institute of Clinical Sciences, Sahlgrenska Academy University of Gothenburg Gothenburg Sweden; ^4^ Sahlgrenska Sports Medicine Center Göteborg Sweden; ^5^ Mayo Clinic Alix School of Medicine Mayo Clinic Rochester Minnesota USA; ^6^ Department of Orthopaedic Surgery and Traumatology Kantonsspital Baselland Bruderholz Switzerland; ^7^ University of Basel Basel Switzerland; ^8^ Department of Computer Science and Engineering Chalmers University of Technology Gothenburg Sweden; ^9^ Department of Orthopaedic Surgery University Medicine Rostock Rostock Germany; ^10^ Department of Orthopaedic and Trauma Surgery Malteser Waldkrankenhaus Erlangen Erlangen Germany

**Keywords:** artificial intelligence, clinical decision support, multimodal artificial intelligence, orthopaedic surgery, personalised medicine

## Abstract

Multimodal artificial intelligence (AI) has the potential to revolutionise healthcare by enabling the simultaneous processing and integration of various data types, including medical imaging, electronic health records, genomic information and real‐time data. This review explores the current applications and future potential of multimodal AI across healthcare, with a particular focus on orthopaedic surgery. In presurgical planning, multimodal AI has demonstrated significant improvements in diagnostic accuracy and risk prediction, with studies reporting an Area under the receiving operator curve presenting good to excellent performance across various orthopaedic conditions. Intraoperative applications leverage advanced imaging and tracking technologies to enhance surgical precision, while postoperative care has been advanced through continuous patient monitoring and early detection of complications. Despite these advances, significant challenges remain in data integration, standardisation, and privacy protection. Technical solutions such as federated learning (allowing decentralisation of models) and edge computing (allowing data analysis to happen on site or closer to site instead of multipurpose datacenters) are being developed to address these concerns while maintaining compliance with regulatory frameworks. As this field continues to evolve, the integration of multimodal AI promises to advance personalised medicine, improve patient outcomes, and transform healthcare delivery through more comprehensive and nuanced analysis of patient data.

**Level of Evidence:** Level V.

**Abbreviations**
AI: artificial inteligence
CT: computer tomography
ML: machine learning
MRI: magnetic resonance imaging
THA: total hip arthroplasty

## INTRODUCTION

**Figure 1:**
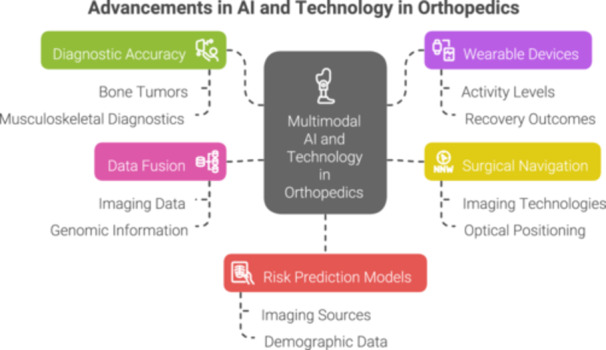
Current technologies in orthopaedic artificial inteligence (AI).

Healthcare is undergoing a transformation driven by multimodal artificial intelligence (AI), which promises to revolutionise how medical data is processed, interpreted and utilised [17]. Multimodal AI represents an approach to data integration, capable of processing multiple types of input simultaneously, including text, visuals, audio, physiological sensors, and environmental cues [10, 17]. This mirrors the way clinicians synthesise various sources of information—such as patient history, imaging, lab results, and real‐time monitoring—to guide decision‐making. However, traditional clinical reasoning relies on human expertise to interpret these inputs, whereas multimodal AI systematically fuses disparate data types within a computational framework. This enables the detection of patterns and interactions beyond human perception, potentially improving prediction accuracy and supporting more data‐driven, personalised care (Figure 1).

The complexity of medical data has long been a challenge in healthcare decision‐making. Multimodal AI addresses this by leveraging advanced computational techniques to analyse heterogeneous data sets that would be difficult for human analysts to comprehend fully [10]. The integration of various data modalities represents a critical advancement in clinical practice, potentially transforming how medical professionals understand and approach patient care. Traditionally, data from different sources was analysed in isolation, limiting the discovery of meaningful correlations and patterns that could enhance medical research and treatment [10, 17]. For example, AI was used to analyse medical registries concerning outcome after anterior cruciate ligament replacement [24]. While decent performance can already be achieved, the clinical use is still limited [24]. The addition of radiographs and possibly activity data (video, continuous monitoring) could further improve outcome prediction. Multimodal AI addresses this limitation by facilitating integrated analysis across varied data types and even secondary sources like patient‐reported outcomes and real‐world data [10, 17].

However, the implementation of multimodal AI is not without challenges. Data availability, privacy concerns, computational infrastructure and regulatory compliance remain significant obstacles [17, 37]. Successful integration requires robust data management practices, sophisticated analytical capabilities, and a multidisciplinary approach that bridges medicine, biology and computer science.

As the healthcare industry continues to evolve, multimodal AI is emerging as a key component in the advancement of personalised medicine, with the potential to enhance drug discovery, patient care and medical research.

## CURRENT STATE OF MULTIMODAL AI IN HEALTHCARE

Multimodal AI can already leverage an extensive range of data sources to create a comprehensive approach to medical analysis. These data types include medical imaging data such as magnetic resonance imaging (MRI) scans, computer tomography (CT) images, radiographs, and ultrasound images [35, 47]. Yoo et al. [47] utilised cranial MRI scans in combination with polygenic risk scores in a multimodal model to differentiate between attention deficit/hyperactivity disorder and typically developing children. Free text and structured data play a crucial role, encompassing electronic health records, clinical notes, laboratory test results, and patient medical histories [19, 29, 30]. The approach extends to omics data, including genomic information, proteomics, metabolomics, and epigenetic data [11].

Physiological and behavioural data further enrich the multimodal AI approach, incorporating information from wearable devices, heart rate monitors, activity tracking, sleep patterns, and continuous glucose monitoring [2, 3, 33]. De Canniere et al. [3] displayed that the combination of traditional cardiac rehabilitation exams combined with continuous monitoring through wearables can be combined into an interpretable machine learning (ML) model. Combining these emerging data sources with traditionally collected metrics of health like patient‐reported outcomes, social determinants of health, and environmental and lifestyle data provides improved context and depth to medical analysis and health predictability [2].

By simultaneously analysing various data sources, AI systems can detect subtle patterns and anomalies that might escape human observation [2, 17, 35]. This comprehensive approach enables more accurate and early disease detection, especially in complex conditions like cancer, neurological disorders, and rare genetic diseases [19, 29, 35, 47]. As demonstrated by Subramanian et al. [35] showing an improvement in predicting lung cancer recurrence when combining thoracic CTs with genomics data.

In the realm of pharmaceutical research, multimodal AI is revolutionising drug discovery and clinical trials [13, 32, 42]. The technology can predict drug interactions, identify promising drug candidates more rapidly, optimise clinical trial design, reduce development time and costs, and enhance the probability of successful drug development [32, 42, 48]. By analysing molecular structures, genetic information, clinical trial results, and patient response data, AI provides unprecedented insights into drug development.

**Figure 2:**
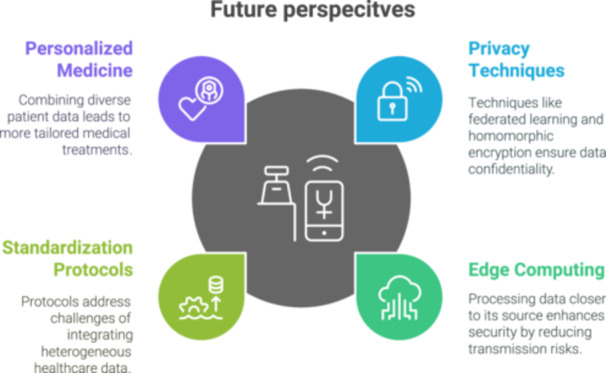
Future perspective of multimodal models in orthopaedics.

Multimodal AI has the potential to advance personalised medicine by enabling the creation of comprehensive patient profiles. By integrating genetic data, clinical histories, lifestyle factors, and real‐world treatment outcomes, healthcare providers may be able to develop more targeted treatment strategies, predict individual patient responses to interventions, reduce the risk of adverse drug reactions, optimise medication dosages, and tailor healthcare approaches to the unique needs of each patient (Figure 2) [2].

## APPLICATIONS IN ORTHOPAEDIC SURGERY

### Presurgical risk prediction

Multimodal AI is revolutionising preoperative planning through sophisticated medical image analysis and predictive modelling. For example, in the domain of bone tumour classification, Song et al. developed the Primary Bone Tumour Classification Transformer Network (PBTC‐TransNet), which addresses a critical challenge in clinical oncology: classifying tumours using incomplete multimodal images [34]. The model demonstrated good performance, achieving a micro‐average AUC (this calculation of area under the curve takes class imbalance into account) of 0.847 in internal testing and 0.782 in external validation.

In a similar approach to developing an ML model based on multiple imaging modalities, He et al. [8] focused on musculoskeletal diagnostics, specifically subscapular tendon injuries. Their multimodal radiomic analysis of shoulder MRI demonstrated significant diagnostic improvements. By extracting 1197 radiomic features across various imaging modalities (T1‐weighted and T2‐weighted coronal, axial, and sagittal images), they achieved a good diagnostic accuracy of 0.867 and an AUC of 0.803 in the external verification group [8]. The study underscores the power of integrating multiple imaging techniques to enhance preoperative assessment accuracy.

In the context of orthopaedic interventions, Liu et al. [21] developed ML models to predict patient outcomes for total knee arthroplasty. Their research revealed that clinical and multimodal data‐driven models could effectively predict postoperative dissatisfaction. The multimodal model combining radiographs with demographics, medical history and preoperative assessments achieved AUC metrics: 0.891 for Knee Society scores, 0.832 for short form‐36 physical component scores, and 0.835 for mental component scores [21]. This approach represents a significant advancement in personalising surgical interventions by identifying patients unlikely to benefit from the procedure.

Geng et al. [6] investigated vertebral compression fracture diagnosis through multimodal MRI‐based radiomics. Their study developed models that could differentiate between benign and malignant vertebral compression fractures with exceptional accuracy. The radiomics model achieved an AUC of 0.905, with an accuracy of 0.817 and sensitivity of 0.831. The multimodal model, utilising XGBoost, demonstrated an AUC of 0.982 with a specificity of 0.979 and positive predictive value of 0.971 [6].

Tiulpin et al. [39] advanced the field of osteoarthritis progression prediction by developing a multimodal ML model. By integrating radiographic data, clinical examinations, and patient medical history, they created a predictive model with an AUC of 0.79 and average precision of 0.68 [39]. This approach significantly outperformed traditional logistic regression methods, offering potential improvements in patient selection for clinical trials and personalised therapeutic planning.

The research by Tong et al. [40] on skeletal bone age assessment further demonstrates the potential of heterogeneous feature learning. Their deep learning model integrated radiographs with additional patient characteristics like race and gender, achieving a mean average error of 0.55 years of bone age assessment [40].

Khosravi et al. [15] built a hip arthroplasty dislocation risk calculator, based on more than 17,000 patients who underwent primary total hip arthroplasty (THA) with a minimum follow‐up of 5 years. Combining preoperative hip radiographs and combining them with patient demographics, the groups model achieved a C index of 0.74 [15]. Utilising Shapley additive explanation, they revealed that their model was primarily driven by preoperative imaging [23].

These studies highlight the potential of multimodal AI in presurgical risk assessment, demonstrating ability to integrate various data sources to enhance diagnostic accuracy and predict surgical outcomes. By leveraging machine learning techniques, these models provide insights that support preoperative planning, patient selection, and personalised treatment strategies. However, while AI‐driven risk prediction may improve decision‐making before surgery, the impact extends beyond preoperative planning to the operating room.

### Intraoperative assistance: Real‐time feedback and guidance during surgery

AI surgical navigation systems represent a sophisticated technological platform designed to enhance surgical precision and outcomes through advanced imaging, tracking, and visualisation technologies. These systems are composed of three essential modules that work in concert to provide surgeons with unprecedented levels of anatomical guidance and instrument control.

The medical imaging module forms the foundational layer of the system, generating high‐resolution examination data that creates detailed three‐dimensional representations of patient anatomy. This module leverages advanced imaging technologies to produce comprehensive anatomical models that serve as the critical reference point for subsequent surgical interventions [1, 9].

The tracking and positioning module represents the dynamic core of the navigation system. This module employs multiple positioning techniques, including ultrasound, optical and mechanical positioning methods [18, 22, 25, 38]. Among these, optical positioning has emerged as the most common approach, particularly in orthopaedic surgical applications. The module integrates specialised sensors typically pre‐fixed to the patient, enabling real‐time tracking of surgical instruments in relation to specific anatomical regions. Sophisticated locators receive and process signals from these sensors, creating a continuous spatial mapping of surgical instruments and target areas.

AI‐driven surgical navigation systems potentially improve intraoperative precision by integrating imaging, real‐time tracking and spatial mapping technologies. These systems enhance surgical accuracy by providing continuous anatomical guidance and instrument positioning, ultimately improving patient outcomes.

### Postoperative care: Continuous monitoring and complication prevention

In the rapidly advancing landscape of medical technology, postoperative care is undergoing a transformation, driven by innovative approaches to patient monitoring and recovery tracking in combination with demographics, imaging and labs. Recent research reveals a compelling narrative of how wearable devices and sophisticated analytics are reshaping our understanding of patient rehabilitation.

Keppler et al. [14] investigated orthogeriatric patients, which starkly illuminated the challenges of postoperative mobility. Their research demonstrated that patients with proximal femur fractures were experiencing extremely limited movement, averaging merely 102.7 steps daily during hospitalisation and that continuous data collection is entirely feasible [14].

Kim et al. [16] explored the potential of wearable technology in spine surgery patients. Their research addressed a long‐standing limitation in medical assessment: the inherent subjectivity of patient‐reported outcomes. By utilising a continuously worn wearable device, they discovered a significant negative correlation between daily steps and pain scores, providing an objective measure of patient recovery that transcends traditional subjective surveys [16].

Scheer and colleagues took this approach further in a multicenter study, demonstrating the feasibility of real‐time physical activity monitoring. Their research showed significant improvements in patient‐reported outcomes, with activity levels providing valuable insights into rehabilitation progress. Importantly, they found correlations between preoperative daily steps and disability indices, suggesting that early activity tracking could potentially predict recovery trajectories.

The comprehensive study by Stienen et al. provided additional depth to our understanding, tracking patient activity for up to one year after spine surgery. Their research revealed a dramatic 71% decrease in activity during the first postoperative week, with recovery occurring gradually over eight weeks. Notably, they also identified that factors such as age, sex, and surgical approach significantly influenced patient activity levels.

Toogood et al. expanded this research into THA, demonstrating the broader applicability of continuous monitoring. Their study tracked 33 patients over 30 days, revealing a steep increase in daily steps from 235 to 2563, with patients being discharged to home showing a significantly higher step count [41].

These studies collectively paint a powerful picture of the future of postoperative care. By leveraging wearable technologies and sophisticated data analysis, healthcare providers can move beyond reactive treatment to a more proactive, personalised approach to patient rehabilitation. The integration of objective, continuous monitoring promises to revolutionise our understanding of recovery, ultimately improving patient outcomes and quality of care.

## TECHNICAL AND ETHICAL CHALLENGES

### Data integration and standardisation

The complexity of data fusion in multimodal modes is underscored by three primary integration approaches: early, intermediate, and late fusion, each presenting unique technical challenges [17]. Data fusion combines various data sources to enhance predictive accuracy and robustness in machine learning, though it also increases model complexity and reduces interpretability. Early fusion requires transforming heterogeneous data sources into a unified feature space, often involving complex mathematical techniques [4, 20, 26]. These methods must carefully preserve the inherent informative characteristics of each data type while creating a cohesive representation. Intermediate fusion offers more flexibility, allowing stepwise model architectures that can selectively extract and combine distinguishing features from multiple modalities, creating more expressive representations than individual data sources [36, 46, 47]. Late fusion, resembling ensemble learning, involves training separate models for each data type and then integrating their predictions, which introduces challenges in developing robust aggregation strategies and ensuring consistent performance across various model architectures [5, 28, 45]. Each approach demands sophisticated computational techniques to handle the inherent variability in biological and clinical data sources, requiring advanced ML methods that can effectively capture complex, multidimensional relationships while mitigating potential information loss during the integration process.

### Ethical and privacy concerns

Ethical and privacy challenges in multimodal health AI are increasingly complex, driven by the breadth and depth of data required for research [2, 27]. The integration of various health data sources introduces significant privacy risks, with potential for individual re‐identification even through de‐identified data sets [27]. Existing regulatory frameworks like the US Health Insurance Portability and Accountability Act (HIPAA) and the European Union's General Data Protection Regulation (GDPR) provide foundational protections, but struggle to comprehensively address the nuanced privacy challenges of multimodal AI [2, 7]. To mitigate these risks, researchers have developed sophisticated technical solutions including differential privacy, federated learning, homomorphic encryption, and swarm learning [7, 12, 31, 43, 44, 50]. These approaches aim to enable collaborative model training while preserving individual data confidentiality, often through techniques like cryptographic data obfuscation, decentralised learning protocols, and blockchain‐enabled secure computation [43]. Additionally, emerging technologies like edge computing offer promising avenues for enhancing data security by processing sensitive information closer to its source, thereby reducing transmission risks and maintaining patient confidentiality [49].

## CONCLUSION

The future of healthcare lies in this integrated, data‐driven approach. Ongoing research and collaborative efforts between technology companies, healthcare providers, and regulatory bodies are crucial to addressing existing challenges and realising the full potential of multimodal AI. As the technology continues to evolve, it promises to transform vast, complex data sets into actionable insights that reduce costs and ultimately improve patient outcomes.

## AUTHOR CONTRIBUTIONS

All listed authors have contributed substantially to this work: Felix C. Oettl and Bálint Zsidai performed literature review and primary manuscript preparation. Editing and final manuscript preparation were performed by Jacob F. Oeding, Michael T. Hirschmann, Robert Feldt, Thomas Tischer and Kristian Samuelsson. All authors read and approved the final manuscript.

## CONFLICTS OF INTEREST STATEMENT

Kristian Samuelsson is a Member of the Board of Directors of Getinge AB. The other authors declare no conflicts of interest.

## ETHICS STATEMENT

The authors have nothing to report.

## Data Availability

Data sharing is not applicable to this article as no data sets were generated or analysed during the current study.
